# Alterations in Dynamic Functional Connectivity in Patients with Cerebral Small Vessel Disease

**DOI:** 10.1007/s12975-023-01148-2

**Published:** 2023-03-27

**Authors:** Futao Chen, Qian Chen, Yajing Zhu, Cong Long, Jiaming Lu, Yaoxian Jiang, Xin Zhang, Bing Zhang

**Affiliations:** 1 Department of Radiology, The Affiliated Drum Tower Hospital of Nanjing University Medical School, Nanjing, 210008 China; 2 Medical Imaging Center, Affiliated Drum Tower Hospital, Medical School of Nanjing University, Nanjing, China; 3 Institute of Medical Imaging and Artificial Intelligence, Nanjing University, Nanjing, China; 4 Department of Radiology, Drum Tower Hospital, Clinical College of Nanjing Medical University, Nanjing, China; 5 Jiangsu Key Laboratory of Molecular Medicine, Nanjing, China; 6 Institute of Brain Science, Nanjing University, Nanjing, China

**Keywords:** Dynamic functional connectivity, Cerebral small vessel disease, fMRI, Independent component analysis

## Abstract

Cerebral small vessel disease (CSVD) is a common disease that seriously endangers people’s health, and is easily overlooked by both patients and clinicians due to its near-silent onset. Dynamic functional connectivity (DFC) is a new concept focusing on the dynamic features and patterns of brain networks that represents a powerful tool for gaining novel insight into neurological diseases. To assess alterations in DFC in CSVD patients, and the correlation of DFC with cognitive function. We enrolled 35 CSVD patients and 31 normal control subjects (NC). Resting-state functional MRI (rs-fMRI) with a sliding-window approach and *k*-means clustering based on independent component analysis (ICA) was used to evaluate DFC. The temporal properties of fractional windows and the mean dwell time in each state, as well as the number of transitions between each pair of DFC states, were calculated. Additionally, we assessed the functional connectivity (FC) strength of the dynamic states and the associations of altered neuroimaging measures with cognitive performance. A dynamic analysis of all included subjects suggested four distinct functional connectivity states. Compared with the NC group, the CSVD group had more fractional windows and longer mean dwell times in state 4 characterized by sparse FC both inter-network and intra-networks. Additionally, the CSVD group had a reduced number of windows and shorter mean dwell times compared to the NC group in state 3 characterized by highly positive FC between the somatomotor and visual networks, and negative FC in the basal ganglia and somatomotor and visual networks. The number of transitions between state 2 and state 3 and between state 3 and state 4 was significantly reduced in the CSVD group compared to the NC group. Moreover, there was a significant difference in the FC strength between the two groups, and the altered temporal properties of DFC were significantly related to cognitive performance. Our study indicated that CSVD is characterized by altered temporal properties in DFC that may be sensitive neuroimaging biomarkers for early disease identification. Further study of DFC alterations could help us to better understand the progressive dysfunction of networks in CSVD patients.

**Supplementary Information:**

The online version contains supplementary material available at 10.1007/s12975-023-01148-2.

## Introduction

Cerebral small vessel disease (CSVD) refers to clinical, imaging, and pathological syndromes caused by various etiologies affecting arterioles and their distant branches, capillaries, and venules in the brain [1]. The known causes and risk factors include age, hypertension, branching atherosclerotic disease, cerebral amyloid angiopathy, radiation exposure, immune-mediated vasculitis, certain infections, and some genetic disorders [2]. Certain fluid biomarkers were found to be associated with CSVD, including elevated levels of low molecular weight neurofilament protein (NF-L), metalloproteinase tissue inhibitor-1, metalloproteinase-2, and metalloproteinase-9 in CSVD patients [3]. Noninvasive evaluation of retinal neurons and vessels may also provide novel biomarkers for the diagnosis and staging of CSVD. Abnormal retinal vessel diameter, increased retinal vessel curvature, decreased vascular fractal dimension, and decreased retinal nerve fiber layer thickness may be found in CSVD patients [4]. However, as of now, the diagnosis of CSVD still be based on brain imaging biomarkers. Neuroimaging features of CSVD include white matter hyperintensity (WMH), lacunar infarct (LI), cerebral microbleeds (CMBs), enlarged perivascular space (EPVS), brain atrophy, and recent small subcortical infarct(RSSI) [5]. The pathogenesis of CSVD has not been fully elucidated. Currently, it is thought that CSVD is a dynamic disorder of whole brain function and that abnormal neurovascular unit (NVU) function plays an important role in the pathogenesis [6]. CSVD can cause a series of clinical symptoms, and CSVD is arguably the most prevalent mechanism contributing to cognitive dysfunction, which is characterized mainly by decreases in executive function, information processing speed, and attention function [7]. Small vessel disease (SVD) is considered to be the leading cause of ischemic stroke and the second most common cause of dementia, second only to Alzheimer’s disease, with CSVD accounting for approximately 20% of all strokes, including 25% of ischemic strokes and 45% of vascular dementia cases [8]. CSVD is responsible for a growing heavy mental and economic burden in aging societies [8, 9]. Therefore, accurately identifying abnormal brain functions caused by CSVD may provide an objective basis for early clinical intervention and useful information for the individualized treatment of patients.

Magnetic resonance imaging (MRI) is the preferred and most important imaging method for diagnosing CSVD. However, conventional imaging features show only the tip of the iceberg of the associated brain damage. Resting-state fMRI (rs-fMRI), which reflects the intrinsic activity of the brain, is an effective non-invasive method for exploring the neural mechanisms of neurological diseases [10]. In recent years, the information generated about human brain networks has revolutionized our understanding of neuroscience and advanced our exploration of the pathogenesis of neurological and psychiatric diseases [11]. The brain network perspective indicates that different brain regions, although spatially distant, are structurally and functionally interconnected, and interact to facilitate brain function [12]. Resting-state functional connectivity (rs-FC), which is defined as the pattern of synchronized neuronal activation, indicates information processing and transference across functionally coordinated brain networks [13]. Several previous studies have revealed reduced FC between the sensory-motor network (SMN) and the visual network (VIS) [14], attenuated FC in the frontoparietal networks [15], increased FC between the right calcarine cortex and the left parahippocampal gyrus (PHG), increased FC in the right inferior temporal gyrus and the left middle temporal gyrus [16], and unvaried difference in FC or brain network topology [17]. Therefore, there is good reason to believe that altered FC can provide a more comprehensive picture of our understanding of brain function transitions, and may be an objective and sensitive imaging marker to identify CSVD.

Most re-FC studies have focused on the assumption that neural activity remains stable during scanning, ignoring the time-varying features of brain functional activity. However, some scholars have shown that brain activity is an inherently dynamic system and that the strength and directionality of FC can change significantly on rapid time scales as discrete FC patterns switch rapidly [18]. Dynamic functional connectivity (DFC) is a new concept that focuses on the dynamic features and patterns of brain networks and has received increasing attention [19]. Numerous studies suggested that this dynamic approach represents a powerful tool to gain novel insight into neurological diseases, such as autism spectrum disorders [20], schizophrenia [21, 22], depressive disorder [23], Parkinson’s disease [24], and Alzheimer’s disease [25]. Group independent component analysis (ICA) and sliding window methods are widely used in DFC analysis [26]. However, to the best of our knowledge, the specific characteristics of the DFC of CSVD are unknown.

In this study, we used group ICA and sliding window methods to investigate the alterations in DFC in CSVD and compare the differences in temporal properties between CSVD and normal control subjects (NC) for early disease identification. We hypothesized that altered DFC temporal properties would be observed in CSVD and that some significant DFC properties may correlate with cognitive function.

## Methods

### Participants

This study was designed following the Declaration of Helsinki and approved by the Research Ethics Committee of Nanjing Drum Tower Hospital. After providing a detailed description of the study procedures to the patients, written informed consent was obtained from each participant. Subjects were enrolled if they met the following criteria: (1) presence of risk factors or family history of cerebrovascular disease, or clinical manifestations of CSVD with age; (2) presence of confluent white matter hyperintensity (Fazekas score ≥ 2) [27, 28], and/or at least one of the following five imaging markers: lacunar infarct (LI), cerebral microbleeds (CMBs), enlarged perivascular space (EPVS), brain atrophy, or recent small subcortical infarct (RSSI); (3) 50–80 years of age; and (4) no cerebral hemorrhage or cerebral infarction. Subjects with the following were excluded: (1) cognitive impairment due to other neurological diseases; (2) cognitive impairment due to other system diseases; (3) severe disease with tumor cachexia; (4) neuropsychiatric diseases such as depression, anxiety, or hysteria; or (5) MRI contraindications.

According to the above inclusion and exclusion criteria, 35 CSVD patients and 31 NC subjects were included in this study. We recorded all demographic and vascular risk factors.

### Neuropsychological assessment

All subjects underwent a standardized neuropsychological test battery performed by an experienced psychologist. We assessed the following six cognitive domains: (1) general cognitive function evaluation using the mini-mental state examination (MMSE) and the Montreal Cognitive Assessment (MoCA); (2) episodic memory measured with the auditory verbal learning test (AVLT), including immediate memory, short-delayed memory, long-delayed memory, cued recall, and recognition; (3) executive function with the Alternative Shape Trail Making Test (TMT-A and TMT-B); (4) language function assessed by the Boston Naming Test and the Animal Verbal Fluency Test (BNT and AFT); (5) processing speed tested with the symbol digit modalities test (SDMT); (6) visuospatial ability assessed by the clock drawing test (CDT).

### Image acquisition

fMRI image data were acquired using a 3 T MRI system (Achieva 3.0 T CX, Philips Medical Systems, Eindhoven, Netherlands) with a 32-channel phased-array coil. The detailed fMRI parameters are as follows: section thickness = 4 mm with no section gap, the field of view (FOV) = 192 × 192 mm^2^, matrix = 64 × 64, echo time (TE) = 30 ms, repetition time (TR) = 2000 ms, and flip angle (FA) = 90°, number of slices = 35, voxel size = 3 × 3 × 4 mm with no gap. In total, 230 volumes were obtained. High-resolution T_1_-weighted structural images of each participant were also acquired with the following parameters: 192 sagittal slices, FOV = 256 × 256 mm, slice thickness = 1 mm with no gap, TR = 9.74 ms, TE = 4.60 ms, and voxel size = 1 × 1 × 1 mm.

### rs-fMRI data preprocessing

Preprocessing of the rs-fMRI data was performed using the advanced version of rs-fMRI Data Processing Assistant (DPARSFA, version 5.3, http://www.restfmri.net) [29] based on the MATLAB platform (The Math Works, Inc., Natick, MA, USA). Slice-timing, head motion correction, nuisance regression with adding mean back (white matter and cerebrospinal fluid (CSF) signals and Friston 24 head motion parameters), and spatial normalization to the standard Montreal Neurological Institute (MNI) EPI template were performed sequentially with a resampled voxel size of 3 × 3 × 3 mm conducted for the 230 time-points. Then, all images were smoothed with a 6 mm full-width at half-maximum (FWMH) Gaussian kernel. Realignment parameters were checked, and two participants showed displacement greater than 3.0 mm or angular rotation more than 3.0°. Thus, we excluded these two subjects from the study. Two-sample *t*-tests indicated no significant differences in the mean framewise displacement (Jenkinson) between the CSVD and NC groups (0.12 ± 0.05 vs 0.12 ± 0.06 mm, *p* = 0.856).

### Group independent component analysis

After rs-fMRI data pre-processing, spatial ICA was conducted to parcellate the data of all subjects with Group ICA of fMRI Toolbox (GIFT) (mialab.mrn.org/software/gift/) [30], and the participant data were decomposed into seven functional networks exhibiting unique time course profiles. This was achieved by using subject-specific and group-level data reduction steps.

First, principal component analysis was applied to reduce the subject-specific data into 120 principal components, and the subject-reduced data were concatenated across time. Next, the data were decomposed into 100 components with the expectation–maximization algorithm at the group level included in GIFT [31]. The Infomax ICA algorithm was repeated 20 times in ICASSO to ensure estimation stability and reliability (http://research.ics.tkk.fi/ica/icasso/) [32]. Finally, participant-specific spatial maps and time courses were obtained with the GICA back reconstruction approach [33].

**Fig. 1:**
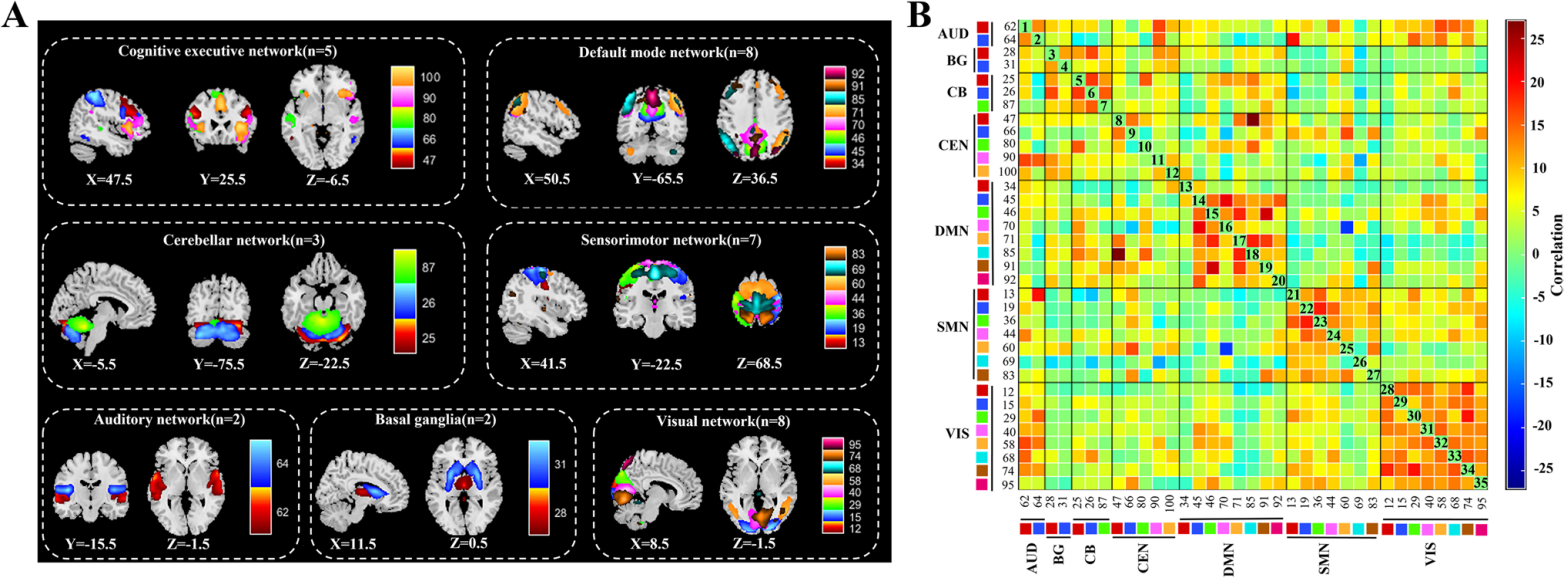
Thirty-five independent components identified by group independent component analysis. **A** Independent component space maps divided into seven functional networks based on their anatomical and functional properties. **B** Group averaged static functional connectivity matrix between pairs of independent components computed using whole resting-state data. AUD, auditory; BG, basal ganglia; CB, cerebellar; CEN, cognitive executive; DMN, default mode; SMN, sensorimotor; VIS, visual

According to a previously described procedure [34], we identified seven functional networks with several independent components (ICs) among the resulting 100 components decomposed by group-level data. We selected the ICs that had peak activations in gray matter showing low spatial overlap with known vascular, ventricular, motion, and susceptibility artifacts, and that exhibited primarily low-frequency power (ratio of power 0.10 to 0.15–0.25 Hz) as meaningful components [35]. Finally, based on the spatial correlation values between the components and the network template [36], the remaining 35 ICs were sorted and rearranged into the seven functional networks shown in Fig. 1: BG, basal ganglia network (ICs 28 and 31); AUD, auditory network (ICs 62 and 64); VIS, visual network (ICs 12, 15, 29, 40, 58, 68, 74, and 95); SMN, sensorimotor network (ICs 13, 19, 36, 44, 60, 69, and 83); CEN, cognitive executive network (ICs 47, 66, 80, 90, and 100); DMN, default mode network (ICs 34, 45, 46, 70, 71, 85, 91, and 92); and CB, cerebellar network (ICs 25, 26, and 87).

Before FC calculation, the following additional postprocessing steps were performed for the time courses of selected ICs: (1) detrending linear, quadratic, and cubic trends; (2) despiking detected outliers with AFNI’s 3dDespike algorithm; and (3) lowpass filtering with a cut-off frequency of 0.15 Hz using a fifth-order Butterworth filter.

### Dynamic functional connectivity analysis

#### Sliding time window analysis

The sliding window approach which is the most common method for investigating DFC was used for the DFC analysis. The DFC analysis process was implemented using the DFC network toolbox in GIFT. As described in previous studies [24], the rs-fMRI data were divided into windows of 22 TR (44 s) size with Gaussianσ = 3 TRs with a step of 1 TR size which has been demonstrated to provide a good compromise between the quality of the correlation matrix estimation and the ability to resolve dynamics. A regularized inverse covariance matrix was used to reduce the noise that is caused by covariance estimates on shorter time series [37]. Next, an additional L1 norm for the precision matrix was imposed to upgrade sparsity in a graphical LASSO framework with 100 repetitions [38]. After calculating the DFC, all FC matrices were converted to *z*-scores using Fisher *z*-transform to stabilize the variance before further analysis.

#### Clustering analysis and calculation of temporal properties and FC strength

We used all window FC matrices across all participants to estimate DFC states. The *k*-means cluster analysis was repeated 100 times, and the Euclidean distance was used to measure the similarity between the FC matrices and regroup them into different clusters [39]. Four were determined to be the optimal number of clusters according to the elbow criteria [21].

We investigated the temporal properties of DFC states by calculating the following parameters: (1) “mean dwell time” which is defined as the number of consecutive windows belonging to a given state; (2) “fractional windows” which are defined as the number of total windows belonging to a given state; and (3) “number of transitions” which is defined as the number of transitioning between states and represents the reliability of each state. A two-sample *t*-test was used to compute group differences in mean dwell time, fractional windows, and the number of transitions, between age-, gender-, and education-matched NC subjects and CSVD patients.

We estimated the subject-specific centroid corresponding to each group-level state by calculating the median value of each FC matrix for that state. Additionally, we used two-sample independent *t*-tests to compare the FC strength of each state at each unique regional pairing (595 pairings; *P* < 0.01) between groups.

### Statistical analysis

Clinical data were statistically analyzed using SPSS software version 26.0 (Statistical Programme for the Social Sciences, SPSS Inc., Chicago, IL). Two-sample *t*-tests were used to compare age, years of education, and cognitive scores, while gender and risk factors were calculated using chi-square tests. We also calculated the partial correlations between the altered DFC temporal properties and cognitive performances, adjusting for vascular risk factors that were significantly different between the two groups. Based on a previous study [24], each subject’s cognitive tests were converted to *z*-scores using Fisher’s *z*-transform by subtracting the mean values divided by the standard deviation to control for the overall effect. If a cognitive domain included more than two tests, the *z*-score for the cognitive domain was obtained by calculating the mean value of the *z*-scores of these cognitive tests.

## Results

### Participant demographics and cognitive characteristics

A total of 35 NC and 31 CSVD patients were included in our sample. Table 1 shows detailed demographic and clinical information. There were no significant differences between the CSVD and NC groups in terms of gender, age, or years of education. Regarding vascular risk factors, the number of patients with hypertension was significantly higher in the CSVD group than in the NC group (*χ*^2^ = 15.696, *P* < 0.001), while there were no significant differences in diabetes, cardiovascular diseases, dyslipidemia, or smoking among participants. The CSVD and NC groups had comparable results for general cognition, episodic memory, language, and visuospatial domains. However, the CSVD group performed worse than the NC group on TMT-A (*t* = 1.89, *p* = 0.001).

**Table 1:** Demographic and clinical data

Variables	NC (*n* = 35)	CSVD (*n* = 31)	Statistics	*p*
Demographic factors				
Age	62.29 ± 5.256	69.35 ± 5.970	*t* = 5.11	0.226
Sex (M/F)	6/29	10/21	*χ*^2^ = 2.045	0.249
Years of education	11.86 ± 2.788	12.95 ± 2.746	*t* = 1.603	0.863
Vascular risk factors				
Hypertension (*n*, %)	5(14.3)	19(61.3)	*χ*^2^ = 15.696	< 0.001**
Diabetes mellitus (*n*, %)	2(5.7)	4(12.9)	*χ*^2^ = 1.028	0.311
Cardiovascular diseases (*n*, %)	1(2.9)	3(9.7)	*χ*^2^ = 1.343	0.335
Dyslipidemia (*n*, %)	7(20.0)	10(32.3)	*χ*^2^ = 1.292	0.256
Smoking (*n*, %)	4(11.4)	4(12.9)	*χ*^2^ = 0.034	1.000
Neuropsychological tests				
General cognitive performance				
MMSE	28.74 ± 1.379	28.03 ± 1.303	*t* = *− *2.143	0.948
MoCA-B	25.71 ± 2.480	24.94 ± 2.190	*t* = *− *1.345	0.802
*z*-scores	0.20 ± 0.904	− 0.23 ± 0.811	*t* = *− *2.001	0.050
Memory performance				
AVLT immediate	17.26 ± 4.680	15.68 ± 4.238	*t* = *− *1.430	0.541
AVLT short	5.69 ± 2.506	4.00 ± 2.436	*t* = *− *2.763	0.852
AVLT long delayed	5.40 ± 2.810	3.35 ± 2.153	*t* = *− *3.268	0.129
AVLT cued recall	4.89 ± 2.878	3.87 ± 2.349	*t* = *− *1.557	0.221
AVLT recognition	21.69 ± 1.659	20.90 ± 2.329	*t* = *− *1.586	0.031*
*z*-scores	0.24 ± 0.895	− 0.27 ± 0.839	*t* = *− *2.370	0.021*
Executive performance				
TMT-A_trail	50.80 ± 11.844	59.68 ± 24.773	*t* = 1.89	0.001**
TMT-B_trail	139.69 ± 37.349	154.61 ± 45.823	*t* = 1.45	0.329
*z*-scores	− 0.19 ± 0.670	− 0.22 ± 1.118	*t* = 1.830	0.072
Language performance				
AFT	18.43 ± 4.736	18.35 ± 4.930	*t* = *− *0.062	0.804
BNT	25.46 ± 3.705	25.91 ± 3.311	*t* = − 0.190	0.850
*z*-scores	0.15 ± 0.927	− 0.02 ± 0.737	*t* = − 0.152	0.800
Attention performance				
SDMT	39.34 ± 10.737	35.42 ± 9.355	*t* = 1.573	0.667
*z*-scores	0.18 ± 1.058	− 0.20 ± 0.922	*t* = 1.570	0.121
Visual-spatial performance				
CDT	26.43 ± 3.458	26.29 ± 2.957	*t* = *− *0.173	0.304
*z*-scores	0.19 ± 1.085	− 0.02 ± 0.928	*t* = –0.171	0.865

Values are the mean ± standard deviation

*MMSE*, mini-mental state examination; *MoCA*, Montreal cognitive assessment; *AVLT*, auditory verbal learning test; *AFT*, animal fluency test; *BNT*, Boston naming test; *TMT-A*, trail-making test part A; *TMT-B*, trail making test part B; *SDMT*, symbol digit modalities test; *CDT*, clock drawing test

^*^p < 0.05, **p < 0.01

### Dynamic functional connectivity differences

**Fig. 2:**
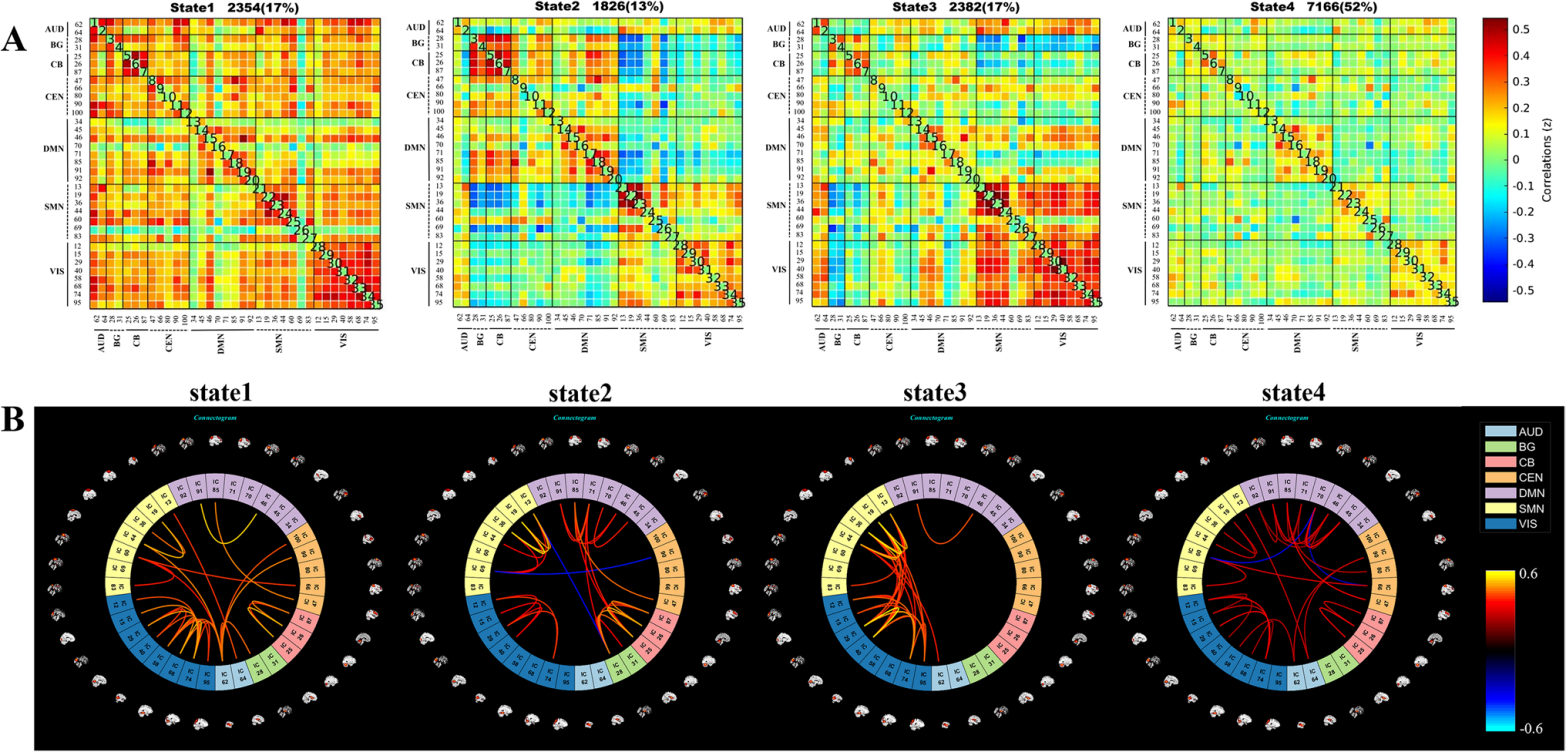
Four distinct functional states identified by *k*-means cluster analysis and corresponding cluster centroids. **A** Group centroid matrices for each state (the number and percentage of total occurrences for each state were listed above each matrix). **B** The 5% strongest functional connections between pairs of independent components in each state. AUD, auditory; BG, basal ganglia; CB, cerebellar; CEN, cognitive executive; DMN, default mode; SMN, sensorimotor; VIS, visual

Four patterns of DFC states of the whole cohort were identified (Fig. 2): (1) state 1, 17% of the windows: a highly connected state of positive intra-network and inter-network coupling involving components of almost the entire brain; (2) state 2 with the lowest frequency: 13% of the windows, distinguished by the predominance of strong positive intra-network FC in the CB, DMN, SMN, and VIS, positive inter-network FC in the BG-CB, and negative correlations in the SMN-BG and SMN-CB; (3) state 3, 17% of the windows: a highly positive inter-network FC in the SMN-VIS and negative correlations in the BG-SMN and BG-VIS; (4) state 4 (with the highest frequency), 52% of the windows: a hyperconnected state with exhibits a sparse connection pattern both intra-network and inter-network for nearly the whole brain.

**Fig. 3:**
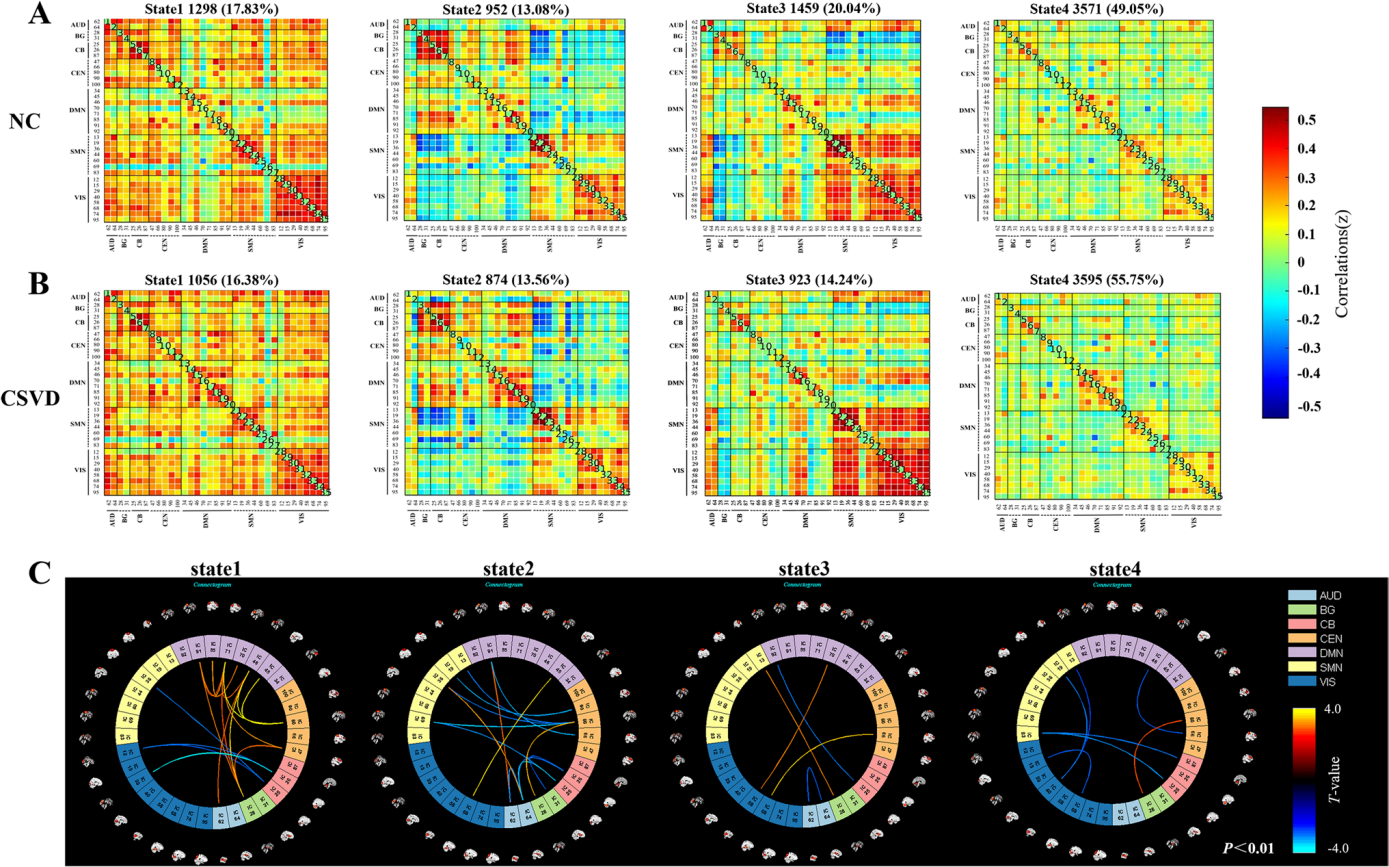
Four dynamic functional connectivity patterns and connectivity strength differences between the two groups. **A** Centroid matrices for the normal control groups. **B** Centroid matrices for the cerebral small vessel disease groups. **C** Stronger and weaker functional connectivity patterns in the CSVD group compared to the NC group (the color bar represents the *T* value, *p* < 0.01). NC, normal controls; CSVD, cerebral small vessel disease

**Fig. 4:**
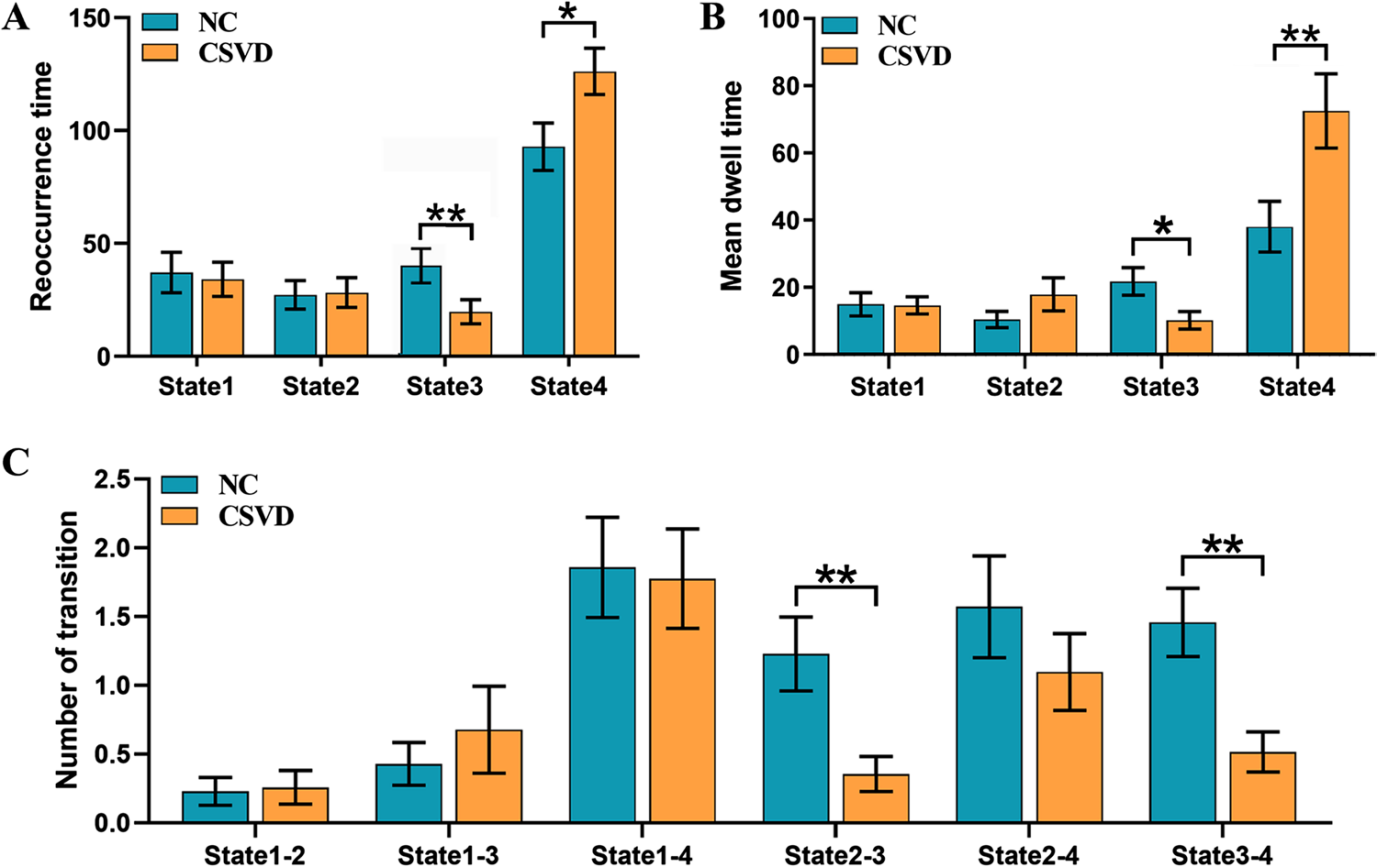
Temporal properties of DFC states between the two groups. **A** Fractional windows in each state. **B** Mean dwell time in each state. **C** The number of transitions between pairs of states. The black lines indicate one standard error above and below the mean. *p < 0.05; **p < 0.05, Bonferroni corrected. NC, normal controls; CSVD, cerebral small vessel disease

The states and group-specific cluster centers of the NC and CSVD groups are shown in Fig. 3A, B, respectively. Chi-square tests showed a significant difference in the proportion of states between the two groups (*χ*^2^ = 98.841, *P* < 0.001). The frequencies of state 2 and state 4 were higher in the CSVD group than in the NC group (state 2 13.56 vs 13.80%; state 4 55.75 vs 49.05%), whereas state 1 and state 3 occurred less frequently in CSVD group than in the NC group (state 1 16.38 vs 17.83%; state3 14.24 vs 20.04%). Regarding differences in time properties, a between-group comparison indicated that the CSVD group had significantly decreased mean dwell times (*t* = 2.361, *p* = 0.022), and fewer fractional windows (*t* = 2.946, *p* = 0.005, Bonferroni corrected) compared to those of the NC group in state 3. In contrast, in state 4, the CSVD group had significantly higher mean dwell times (*t* =  − 2.616, *p* = 0.011, Bonferroni corrected), and more fractional windows (*t* =  − 2.253, *p* = 0.028) than the NC group. In addition, we observed significant reductions in the transitions between state 2 and state 3 (*t* = 2.935, *p* = 0.005, Bonferroni corrected), and between state 2 and state 4 (*t* = 3.280, *p* = 0.002, Bonferroni corrected) in the CSVD group compared to the NC group (Fig. 4).

### FC strength of dynamics states

FC strength for each state was compared between the CSVD and NC groups and is shown in Fig. 3C. For state 1, we observed stronger inter-network FC (DMN-AUD, DMN-BG, and DMN-CEN) and weaker inter-network FC (CB-VIS and CB-SMN) in the CSVD group compared to the NC group. Compared with the NC group, the CSVD group had weaker inter-network FC located mainly in CEN-SMN and CB-AUD in state 2, and weaker inter-network FC, located mainly in SMN-VIS and CB-SMN in state 4. In addition, in state 3, the difference in functional connectivity strength between the CSVD and NC groups was relatively small, we observed stronger inter-network FC (DMN-VIS, SMN-BG, and CEN-VIS), weaker inter-network (CB-DMN), and intra-network (AUD) FC in the CSVD group compared to the NC group. However, there was a significant difference in the strength of FC in each state between the two groups (*p* < 0.01).

### Correlations between DFC properties and cognitive performance

As shown in Table 2, our study revealed that DFC properties were associated with cognitive performance. Specifically, mean dwell time in state 4 was negatively correlated with visual-spatial performance (*r* =  − 0.337, *p* = 0.006). Additionally, fractional windows had a significant positive correlation with general cognitive performance in state 3 (*r* = 0.247, *p* = 0.047). Finally, the number of transitions between state 1 and state 3 was positively correlated with executive performance (*r* = 0.265, *p* = 0.033).

**Table 2:** Significant correlations between dynamic functional connectivity temporal properties and neuropsychological characteristics

			Cognitive domains					
			General cognitive	Memory	Executive	Language	Attention	Visual-spatial
Mean dwell time	State4	*r*	− 0.036	0.013	0.014	0.019	− 0.065	** − 0.337**
		*P*	0.777	0.919	0.911	0.822	0.607	0.006**
Fractional Windows	State 3	*r*	**0.247**	0.068	− 0.093	0.213	0.107	0.150
		*p*	0.047*	0.590	0.416	0.088	0.394	0.235
Number of transitions	Sate 1–3	*r*	0.044	− 0.115	**0.265**	0.160	− 0.007	0.176
		*p*	0.729	0.361	0.033*	0.203	0.953	0.161

A partial correlation test was used. *p* values were adjusted for hypertension. *p < 0.05; **p < 0.01

## Discussion

In the present study, we used the sliding-window approach and *k*-means clustering based on ICA to investigate the dynamic characteristics of DFC in CSVD. Our study revealed these main findings: (1) four distinct connectivity states were identified across the whole cohort and state 4 which is a sparse connection pattern that occurred most often; (2) our results showed altered DFC temporal properties of mean dwell time, fractional windows, and the number of transitions in the CSVD group; (3) FC strength for each state was significantly different between the two groups; and (4) DFC properties for different states exhibited significant correlations with cognitive performance. These findings provided new insight into the role of DFC in elucidating the pathophysiological mechanisms of CSVD.

As described in the “Results” section, we identified four distinct functional connectivity states in our cohort. State 4, which is a hyperconnected state exhibiting a sparse connection pattern both intra-network and inter-network in nearly the entire brain, had the highest frequency (52%). A previous study found that the weakly connected DFC state is associated with self-focused thinking [40]. Moreover, the sparse state profile is considered a baseline connectivity pattern, while other states with strong positive or negative connectivity may reflect the neuropsychological processes of the disease [41]. We observed that state 4 occurred 6.70% more often and state 3 occurred less often in the CSVD group than in the NC group. The higher incidence of state 4 in the CSVD group may indicate that CSVD favors a state with less information transfer but a greater metabolic energy reserve [42]. Our findings were in line with a previous study using cluster analysis to study the temporal properties of patients with CSVD [43]. The complete minimization of wiring would allow only local connections and remote connections, resulting in information transfer delays and metabolic energy depletion [42]. To counteract this effect, the brain also minimizes energy costs by adding several long-distance connections and creating a small-world network [44]. The reduction in “crosstalk” and increased “segregation” between brain networks in CSVD indicates their resting-state network vulnerability which means that the configurations of multiple brain regions interacting in complex and flexible communication patterns may be disrupted in CSVD.

Regarding the temporal properties of DFC, our study found that the CSVD group had significantly fewer fractional windows and shorter mean dwell times in state 3 than the NC group, indicating the disconnection of intra-network FC and the reduced integration of inter-network FC between the SMN and VIS network in the CSVD group. A recent study showed that the increase in WMH burden in patients with CSVD was associated with a decrease in time spent in high-occupancy states [45]. Despite slight differences in inclusion criteria, the results showed similarities. Previous evidence suggested that damage to the white matter of the brain in CSVD leads to extrapyramidal syndrome accompanied by dominant posture, gait disturbances, and visuospatial dysfunction [46, 47]. More importantly, gait disturbances and visuospatial dysfunction are important hallmark features of CSVD and the most common causes of falls and disability. It is recognized that visuospatial and vestibular functions are essential for the maintenance of gait and balance [48]. FC in the supplementary motor area (SMA) which is an important part of the SMN was decreased in CSVD with gait disorders compared with CSVD without gait disorders [49]. Our results provided evidence for the mechanism of visuospatial dysfunction and gait instability in CSVD patients, namely, the reduction in FC between the SMN and VIS network.

In addition to these findings, the CSVD group also had significantly more fractional windows and longer mean dwell times in state 4 than the NC group. Our data are consistent with previous studies on many other neurological and psychiatric diseases, including Alzheimer’s disease [42], Parkinson’s disease [24], and schizophrenia [21]. Another study had also shown that spending more time in a weakly connected state is associated with insufficient cognitive reserve, which may reflect a lack of network efficiency [19]. CSVD is the most common of the mechanisms involved in vascular cognitive impairment [50]. Recent research had demonstrated that the mechanisms leading to cognitive declines caused by CSVD include blood–brain barrier dysfunction, reduced cerebrovascular reactivity, and impaired perivascular clearance [51]. Evidence suggested that CSVD also affects cognition by disrupting structural and functional networks, leading to disconnection syndrome [52]. Our findings explain the mechanism by which CSVD causes cognitive impairment at a macroscopic level.

In addition, our study demonstrated a significant reduction in the number of transitions between state 2 and 3, and between 3 and state 4 in the CSVD group compared to the NC group. State transitions are thought to reflect the metastability of neural activity, which allows multiple brain functional networks to be flexibly integrated and separated in coordination without being locked into a fixed interaction pattern. Frequent transitions between discrete connectivity modes also facilitate flexible information integration and intensive information exchange across multiple networks [53]. A study of the Philadelphia Neurodevelopmental Cohort showed that the probability of transitions between states is limited by the linear spread of activity along structural connectomes [54]; thus, reduce in the number of transitions in CSVD patients indicated that multiple brain regions integrating and interacting with complex information are disrupted, possibly due to the destruction of white matter fibers by WMH.

Regarding the correlation analyses of the DFC properties and cognitive performance, we observed that the worsening of visual-spatial performance correlated with increased dwell time in state 4. State 4 is a weak connection pattern both intra-network in VIS and inter-network between VIS and other functional networks; this resulted in worse visual-spatial performance in the CSVD group. Additionally, more fractional windows in state 3 predicted better general cognitive performance. In our study, general cognitive performance was assessed using MMSE and MoCA which are currently the most widely used cognitive function screening tools in clinical practice; these tools are used mainly for the initial screening of various types of neurodegenerative diseases [55]. Our results indicated that the fractional windows of DFC could be thought of as an imaging marker that predicts the occurrence of CSVD. Finally, we observed a significant positive association between the number of transitions between state 1 and state 3 and executive performance. A previous study found that better executive performance was associated with more frequent connection states [56]. Frequent state transitions are associated with metastable properties, and cognitive flexibility may be facilitated by greater variability in functional connections. These findings suggested that improved executive performance may be related to a tendency to occupy more frequently occurring brain configurations that enable cognitive flexibility. Thus, the altered DFC properties may be potential imaging biomarkers that can aid in the clinical diagnosis of CSVD.

We acknowledge several limitations of the present work. First, though CSVD is a progressive disease, our study is a small-sample cross-sectional study. Therefore, large-sample longitudinal studies will be needed to explore the relationship between DFC properties and CSVD. Additionally, the CSVD patients in our study were not further grouped according to disease severity, which could have unpredictable effects on the present results. To elucidate the relationship between DFC properties and disease more accurately, further classification of CSVD based on total MRI brain small vessel disease burden is necessary. Finally, previous studies had suggested that the rs-fMRI acquisition time should be greater than 10 min to meet standards for DFC analysis, but the rs-fMRI scan lasted only 8 min for each subject in our study. Future studies with a longer scanning time are needed to verify our findings.

## Conclusion

In summary, we investigated alterations in DFC temporal properties in CSVD. We found decreased mean dwell times and fractional windows in state 3, increased mean dwell time fractional windows in state 4, and reductions in transitions between state 2 and state 3, and between state 2 and state 4 in the CSVD group compared to the NC group. Furthermore, some altered time-varying metrics were associated with cognitive performance. Our study indicated that CSVD may be characterized by altered temporal properties in DFC, which may be sensitive neuroimaging biomarkers for early disease identification. Further studies on alterations in DFC could help us to better understand the progressive dysfunction of networks in CSVD patients.

### Supplementary Information

Below is the link to the electronic supplementary material.

Supplementary file1 (DOCX 19 KB)

## Data Availability

Due to the clinical nature of the data, the data that support the findings of this study are not freely available but can be made available by the corresponding author, upon reasonable request. A formal data-sharing agreement is needed before any data can be shared.

## Declarations

### Disclaimer

The funders had no role in the study design, data collection and analysis, decision to publish, or preparation of the manuscript.
