# Supplementary material for: Alterations in Dynamic Functional Connectivity in Patients with Cerebral Small Vessel Disease
Source: Transl Stroke Res. 2023 Mar 27;15(3):580–90. doi: 10.1007/s12975-023-01148-2 (PMC11106163; doi:10.1007/s12975-023-01148-2)
Supplement: Supplementary file 1 — Supplementary file1 (DOCX 19 KB) [file 12975_2023_1148_MOESM1_ESM.docx]

**SUPPLEMENTAL TABLE 1 | Dynamic functional connectivity temporal properties**

|  |  | **NC(n=35)** | **CSVD(n=31)** | **Statistics** | ***p*** |
| --- | --- | --- | --- | --- | --- |
| Fractional windows (%) | State 1 | 1298(17.83) | 1056(16.38) | *χ*^2^=98.841 |  |
|  | State 2 | 952(13.08) | 874(13.56) |  |  |
|  | State 3 | 1459(20.04) | 923(14.24) |  | **＜0.001**** |
|  | State 4 | 3571(49.05) | 3595(55.75) |  |  |
| Fractional windows | State 1 | 37.09±53.322 | 34.06±42.482 | *t(64)=*0.256 | 0.799 |
|  | State 2 | 27.20±37.652 | 28.19±36.629 | *t(64)=*0.109 | 0.914 |
|  | State 3 | 41.68±53.510 | 29.77±29.989 | *t(64)=*2.946 | **0.005**** |
|  | State 4 | 102.03±62.644 | 115.97±57.507 | *t(64)=*-2.253 | **0.028*** |
| Mean dwell time | State 1 | 14.98±20.758 | 14.64±14.375 | *t(64)=*0.078 | 0.938 |
|  | State 2 | 10.43±14.444 | 17.89±27.574 | *t(64)=*-1.398 | 0.167 |
|  | State 3 | 21.73±24.393 | 10.17±14.673 | *t(64)=*2.361 | **0.022*** |
|  | State 4 | 38.00±44.878 | 72.47±61.665 | *t(64)=*-2.616 | **0.011**** |
| Number of transitions | State 1-2 | 0.23±0.598 | 0.26±0.682 | *t(64)=*-0.187 | 0.852 |
|  | State 1-3 | 0.43±0.917 | 0.68±1.759 | *t(64)=*-0.733 | 0.466 |
|  | State 1-4 | 1.86±2.158 | 1.77±2.012 | *t(64)=*0.161 | 0.873 |
|  | State 2-3 | 1.23±1.592 | 0.35±0.709 | *t(64)=*2.935 | **0.005**** |
|  | State 2-4 | 1.57±2.187 | 1.10±1.557 | *t(64)=*1.024 | 0.310 |
|  | State 3-4 | 1.46±1.462 | 0.52±0.811 | *t(64)=*3.280 | **0.002**** |

*Values are the mean ± standard deviation. *p < 0.05,* ***p < 0.05,* *Bonferroni correction.*
